# Perinatal Diagnosis and Management of a Case with Interrupted Aortic Arch, Pulmonary Valve Dysplasia and 22q11.2 Deletion: A Case Report

**DOI:** 10.3390/medicina59101838

**Published:** 2023-10-16

**Authors:** Radu Vlădăreanu, Călina Maier, Raluca Tocariu, Marcela Șerban, Elvira Brătilă

**Affiliations:** 1 Department of Obstetrics And Gynecology, Elias University Emergency Hospital, 011461 Bucharest, Romania; vladareanu@gmail.com; 2 Department of Obstetrics And Gynecology, University of Medicine and Pharmacy “Carol Davila”, 020021 Bucharest, Romania; elvirabarbulea@gmail.com; 3 Department of Obstetrics And Gynecology, Clinical Hospital of Obstetrics and Gynecology “Prof. Dr. Panait Sîrbu”, 060251 Bucharest, Romania; tocariuraluca@yahoo.com (R.T.); marcelaserban83@yahoo.com (M.Ș.)

**Keywords:** prenatal, IAA, ventricular septal defect, pulmonary dysplasia, ARSA, 22q11.2DS

## Abstract

The 22q11.2 deletion syndrome (22q11.2DS) is the most common chromosomal microdeletion disorder caused by hemizygous microdeletion of the long arm of chromosome 22. It is now known to have a heterogenous presentation that includes multiple additional congenital anomalies and later-onset conditions, such as gastrointestinal and renal abnormalities, autoimmune disease, variable cognitive delays, behavioral phenotypes and psychiatric illness. The purpose of our paper is to present the case of a fetus diagnosed with a complex association of cardiac anomalies: interrupted aortic arch type B, large malalignment-type ventricular septal defect, pulmonary valve dysplasia, and aberrant right subclavian artery for whom the result of genetic testing revealed 22q11.2 deletion. The pregnancy was regularly followed until delivery which took place in Germany so that neonatal cardiac surgery could be performed in an experienced center for cardiac malformations. The distinctivness of our report resides in the fact that it offers a complete image of a case of 22q11.2 deletion syndrome starting from the prenatal diagnosis (and emphasizing on the most relevant sonographic features) and, with parents not opting for termination of pregnancy, ending with the newborn surviving major cardiac surgery, offering thus the possibility to bring into focus postnatal outcome and future expectations in similar cases.

## 1. Introduction

Interrupted aortic arch (IAA) is a rare, severe form of congenital heart defect (CHD) characterized by complete anatomical discontinuity between two adjacent segments of the aortic arch [1]. IAA occurs in about 1% of CHD and is classified as type A, B and C based on the anatomic location of the interruption in relation to the brachiocephalic vessels, being rarely diagnosed in fetal series. In type A, the disruption is located distal to the left subclavian artery, in type B, between the left carotid artery and the left subclavian artery and in type C, the disruption is located between the innominate artery and left carotid artery, this being the rarest type [2].

Although clinically under recognized, 22q11.2DS is the most common microdeletion syndrome with an estimated prevalence of 1 in 4000 live births [3,4]. The first description in the English literature of the constellation of findings now known to be due to this chromosomal difference was made in the 1960s in children with DiGeorge syndrome, who presented with the clinical triad of immunodeficiency, hypoparathyroidism and congenital heart disease [5]. The phenotypic spectrum of the 22q11.2DS includes a wide variety of malformations and abnormalities occurring in different combinations and with widely differing severity [4].

In 20% of malformations of the outflow tract, and in IAA type B, 22q11.2DS occurs. This association is significantly predicted by the presence of associated ultrasound findings: thymic hypo/aplasia, intrauterine growth restriction (IUGR) and additional aortic arch anomalies [6]. This shows that IAA type A and type B are distinct entities and while in IAA, type B in more than 50% of cases 22q11.2DS may be expected, type A is not commonly associated with 22q11 hemizygosity (as reported in Volpe’s paper, in only one case out of three of IAA type A FISH analysis detected 22q11.2 microdeletion) [7].

Screening options for 22q11.2DS include noninvasive prenatal testing (NIPT) and imaging. NIPT has a 70–83% detection rate and a 40–50% positive predictive value and prenatal imaging, usually by ultrasound, aims to detect several physical features associated with the syndrome [8]. Definitive diagnosis is made by genetic testing of chorionic villi or amniocytes using a chromosomal microarray. About 85–90% of 22q11.2 microdeletions arise as de novo events and, in contrast to trisomies, are not related to parental age. The 22q11.2 region of the human genome facilitates the potential for non-allelic unequal homologous crossover (recombination) during meiosis, which can result in microdeletions [8].

Taking all of the above into account, we draw attention to the case of an association of 22q11.2 deletion with not only one, but a complex of cardiac anomalies (IAA type B, large malalignment-type VSD, pulmonary valve dyplasia and ARSA) that we followed all the way from the moment of the prenatal diagnosis until delivery, major cardiac repair surgery and 4 weeks post surgery. What brings uniqueness to our paper is the fact that with parents not deciding to terminate the pregnancy, we have the opportunity to describe in detail the evolution of such a case, stressing the importance of correct counselling after announcement of the diagnosis. Moreover, we address the topic of extened panel NIPT (including microdeletions) in view of the possible different approach to our case should this test had been performed initially. 

## 2. Case Report

We present the case of a 34-year-old IGIP who obtained a spontaneous pregnancy while starting diagnostic work-up for infertility and was examined in our department for the second trimester anomaly scan. The first trimester anomaly scan was reported as normal and she had a low-risk NIPT result for trisomies 21, 18 and 13. Her medical and family history were uneventful. At 22 weeks, the ultrasound differential diagnosis of CHD was made between severe aortic coarctation and tubular hypoplasia with VSD and IAA. A dilated pulmonary artery was also revealed. The fetal echocardiography performed by a pediatric cardiologist established the final diagnosis as IAA type B with VSD, a pulmonary valve dysplasia without fulfilling the criteria of pulmonary stenosis (with a peak velocity in the pulmonary trunk of 98 cm/s, a value which is situated above the 95 percentile) and ARSA. The z-score for the aortic root at 22 weeks was −1.65.

**Figure 1:**
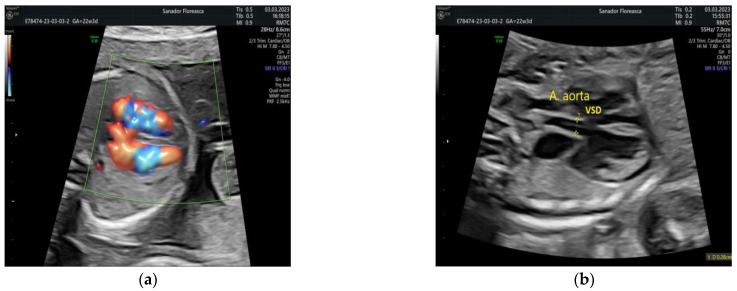
Four-chamber view in Color Doppler (**a**) showing a slight ventricular disproportion and the perimembranous VSD. Five-chamber view (**b**) showing in addition to the VSD, a small ascending aorta. VSD, ventricular septal defect.

The four-chamber view showed a slightly ventricular disproportion with a narrow left ventricle when compared to the right ventricle (with a right ventricular/left ventricular diameter ratio of 1.3), similar to findings in fetuses with aortic coarctation and the perimembranous ventricular septal defect. The five-chamber view showed a perimembranous VSD and a small descending aorta (Figure 1).

**Figure 2:**
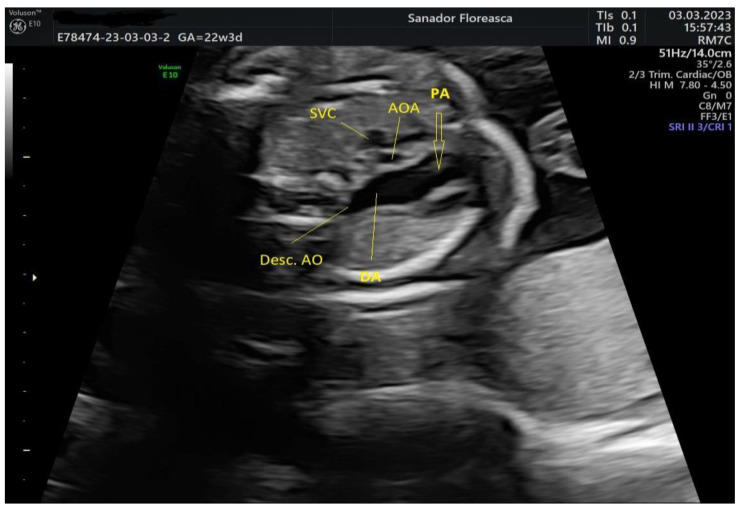
Three-vessel-trachea view. The continuity of the ascending aortic arch (AoA) toward a transverse aortic arch cannot be seen and the trachea is adjacent to the dilated pulmonary artery (PA) and ductus arteriosus (DA). PA, pulmonary artery; AoA, ascending aortic arch; SVC, superior vena cava; Desc AO, descending aorta; DA, ductus arteriosus.

The three-vessel-trachea view revealed one of the main diagnostic features of IAA which is the lack of continuity of the aortic arch; moreover, the trachea appeared in close proximity with the pulmonary artery because of the absence of the medially located aortic arch and the pulmonary trunk was dilated (Figure 2). 

**Figure 3:**
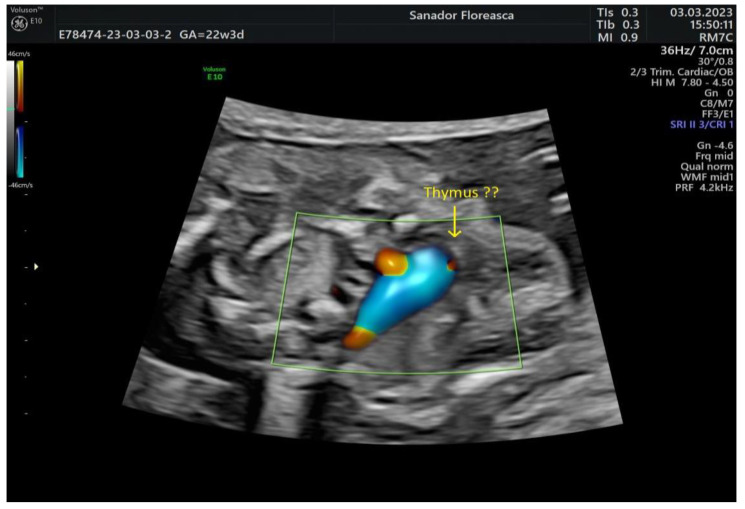
Three-vessel-trachea view in Color Doppler. Again, the continuity of the ascending aortic arch with the descending aorta cannot be demonstrated and the trachea is close to the dilated pulmonary artery. Note the presence of the hypoplastic thymus. ??: severe hypoplastic thymus.

The thymus appeared to be at least hypoplastic and, in consequence, the pulmonary artery was noted to be in close proximity to the sternum anteriorly (Figure 3). The thymic-to-thoracic ratio was 0.3. 

A more clear picture of the cardiac anomalies reported can be seen in Video S1.

Invasive genetic testing (amniocentesis with microarray) revealed 22q11.2 microdeletion and genetic counselling was offered to the couple, including parental genetic studies to investigate wether 22q11.2 microdeletion was de novo or not, but the parents refused them. In addition, they received counselling for future pregnancies. Accordingly, the patient decided to continue the pregnancy. She had regular follow-ups with ultrasound scans every 2–3 weeks to monitor fetal growth and condition and amniotic fluid volume.

**Figure 4:**
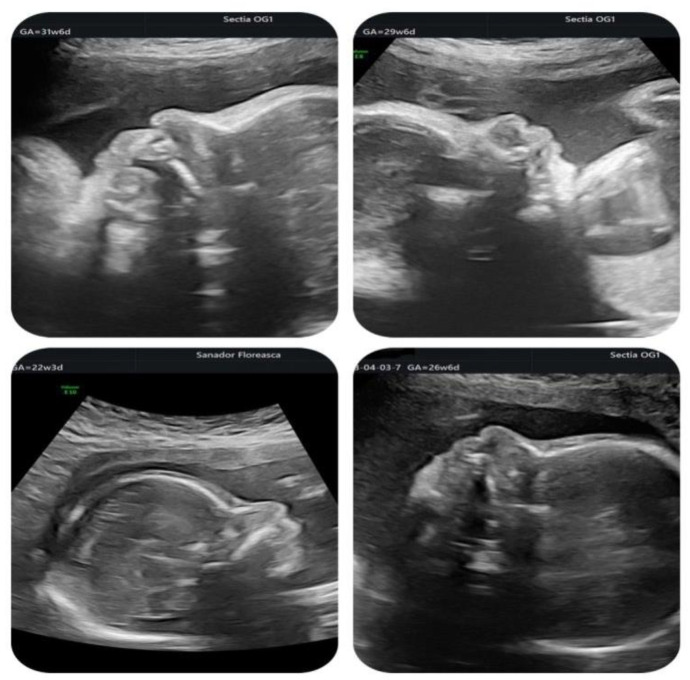
Fetal profile at different gestational ages showing a bulbous nose.

In the second half of gestation, we observed an enlarged cavum septum pellucidum (CSP) and polyhydramnios. The corpus callosum looked normal. Fetal profile images at various gestational ages throughout the pregnancy revealed a bulbous nasal tip which is frequently associated with 22q11.2 DS, but without being pathognomonic (Figure 4).

**Figure 5:**
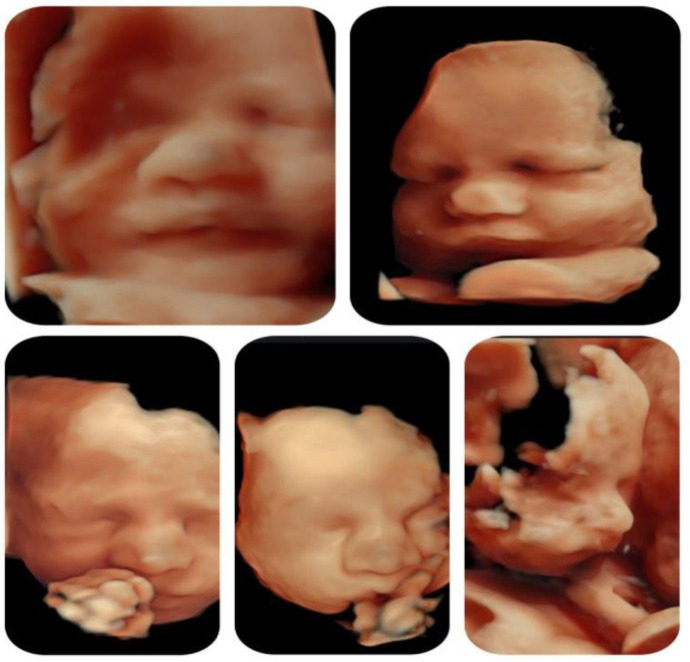
3D reconstruction of fetal face at different gestational ages. Except for the fetal profile showing the bulbous nose, the face appears normal.

Although it is known that facial dysmorphism is typical in infants with this microdeletion, the prenatal ultrasound signs are not specific and can be subjective. The 3D reconstruction of fetal did not show any particular features throughout the gestation with exception of the bulbous nose (Figure 5).

The patient delivered vaginally at 40 weeks in a tertiary center with immediate availability of pediatric cardiology services. The female newborn weighed 3420 g, measured 48 cm and received an Apgar score of 8/9/10. The echocardiography performed after birth confirmed the cardiac anomalies. The VSD measured 8–10 mm and spectral Doppler showed a maximum speed across the pulmonary valve of 150 cm/s. During her stay in the cardiology unit, the baby was stable and received prostaglandins through a Swan–Ganz catheter.

After 7 days, the baby underwent complex cardiac corrective surgery with median sternotomy approach: extended latero-terminal anastomosis of the aortic arch, closure of VSD with bovine pericardial patch and closure of the patent ductus arteriosus. She remained intubated for 5 days after surgery and received a 10-day course of antibiotics and anticoagulant medication (heparin initially followed by acetylsalicylic acid (ASA)). Following an uneventful recovery and with a satisfactory postoperative result with good biventricular circulation, the newborn was discharged after 2 weeks. The cardiology team recommended sternal precautions for 10 weeks, one-year prophylactic therapy with ASA and endocarditis prophylaxis and regular check-up examinations with the pediatrician.

At 7 weeks after birth, the baby’s evolution is favorable and she is undergoing follow-up in a multidisciplinary team (pediatrician, pediatric cardiologist, genetics specialist, family physician).

The patient’s characteristics are summarized in Table 1.

Informed consent from the patient regarding the use of information about the diagnosis and also the ultrasonographic material and evolution of the pregnancy and of the newborn was obtained.

## 3. Discussion

Despite widely available genetic testing for nearly 30 years, 22q11.2DS remains under diagnosed, in part due to its multisystem nature and variable severity of associated features [5,8,9]. Although common, lack of recognition of the condition and/or lack of familiarity with genetic testing options, together with the wide variability of clinical presentation, delays diagnosis. Early diagnosis, preferably established prenatally could improve outcome and prognosis and allow a more appropiate counselling of the parents.

Significant progress has been made in understanding the complex molecular genetic etiology of 22q11.2DS underpinning the heterogeneity of clinical manifestations. The deletion is caused by chromosomal rearrangements in meiosis and is mediated by non-allelic homologous recombination events between low-copy repeats or segmental duplications in the 22q11.2 region. A range of genetic modifiers and environmental factors, as well as the impact of hemizygosity on the remaining allele contribute to the intricate genotype-phenotype relationships [10].

For couples with no history of 22q11.2 microdeletion diagnosis in either parent, there are several possible routes to prenatal diagnostic testing that could identify a fetus with 22q11.2DS [8]. 

Although the diagnosis of this microdeletion has been traditionally based on the recognition of clinical features and cytogenetic testing using the fluorescence in situ hybridization (FISH) technique, poor clinical accuracy, the low confirmatory rate in the screening of suspected microdeletions and failure to detect other than the targeted microdeletion are the major drawbacks of this method [10]. So, the golden standard method for detecting 22q11.2 microdeletions remains chromosomal microarray performed in invasively obtained prenatal samples, such as chorionic villi and amniotic fluid, as it was in our case. 

A multicenter prospective observational study published recently evaluated the performance of single nucleotide polymorphism (SNP)-based noninvasive prenatal testing (NIPT) for the 22q11.2 microdeletion in a large cohort of women. The results showed that noninvasive cell-free DNA prenatal screening can detect most affected cases, including smaller nested deletions, with a low false positive rate. The reported sensitivity of the test was 75%, specificity of 99.84%, positive predictive value of 23.7% and negative predictive value of 99.98% [11]. Other studies revealed also that prenatal screening for 22q11.2 deletion has become clinically feasible, with an analytical sensitivity of 69.6% and 75.4%, respectively [12,13]. Our patient had a standard panel NIPT performed in the first trimester, this raising the question wether the approach could have been different should an abnormal result for 22q11.2 microdeletion had been obtained before the ultrasonographic diagnosis of the anomalies.

The most frequent association is made between 22q11.2DS and congenital heart disease and cleft palate [14]; moreover, cardiac anomalies reported include conotruncal anomalies such as interrupted aortic arch, common arterial trunk, absent pulmonary valve syndrome, pulmonary atresia with VSD, tetralogy of Fallot and conoventricular septal defects [15,16]. The presence of a right aortic arch, an aberrant right subclavian artery, especially in combination with a cardiac anomaly, a hypoplastic thymus or other extracardiac anomalies increases the risk for deletion of 22q11.2 [17,18]. 

In our case, the final fetal cardiologic diagnosis was IAA type B with a large VSD, pulmonary valve dysplasia and ARSA. In our opinion, one strong point of the present case report is that it presents a combination of cardiac anomalies that are demonstrated in detail by ultrasonographic pictures, emphasizing not only the severity of the diagnosis, but also the major questions that the practitioners should expect from the parents after announcement of such a diagnosis.

IAA is a rare form of critical neonatal heart disease and in the absence of prenatal diagnosis patients with IAA present in shock when the patent ductus arteriosus closes; thus, initiation of prostaglandin E1 infusion allows for adequate lower body perfusion prior to surgical repair [19]. As a result of accurate prenatal diagnosis, our patient was admitted to a tertiary pediatric cardiology unit where the surgical intervention was performed successfully after receiving adequate prostaglandin therapy.

Moreover, the surgical approach is tipically perfomred by a median sternotomy which results in a large scar needing special care for a minimum of 10 weeks, as it was recommended to our patient. Although to date 22q11.2 DS does not represent an independent risk factor for mortality after cardiac surgery, the short-term surgical outcome may very for subgroups of patients with specific cardiac phenotypes. In particular, the association between the presence of 22q11.2 and prolonged total length of stay, longer time of mechanical ventilation and intensive care and higher frequency of cardiac events were statistically significant in the subgroup of patients with IAA [20]. Also, patients can develop hypocalcemia and may require calcium supplementation. Immunodeficiencies may be associated with the increased rate of postoperative infections and may dictate the need for specific transfusion management practices (transfusion of filtered/irradiated and cytomegalovirus-seronegative blood products) [21]. 

A comparative study showed that neonates with this microdeletion who undergo neonatal cardiac surgery have a significant abnormal neurodevelopmental outcome when evaluated at 18 to 24 months of age compared with a matched group without 22q11.2DS [22]. This information is extremely important in highlighting the need for early targeted medical and rehabilitation intervention through a multidisciplinary approach for these fragile and at-risk patients. This finding stresses one weak point of our paper: it does not offer data regarding the long-term outcome of the patient and assessment of the potential complications.

It is rare for patients with isolated IAA to survive to adulthood without operation; still, cases of isolated IAA diagnosed using CT angiography have been reported in the literature [23,24,25,26]. In a retrospective study published by Jiang et al., it was observed that in these cases the patients had extensive collateral vessels joining the descending aorta. Anti-hypertensive medical management with long-term follow-up appears to be a reasonable treatment option for these patients, although surgical intervention is a good choice [23]. This is not applicable in our case report considering that the fetus was diagnosed with a severe complex of cardiac anomalies and also a genetic syndrome, thus warranting immediate surgical correction.

The age of the newborn to undergo the operation was in our case 7 days, which is consistent to the study reported by Onalan et al. [27]. Yet, 38% of their patients required reintervention due to aortic arch restenosis during the follow-up period. Preoperative aortic root size is a predictive factor for postoperative left ventricular outflow tract obstruction after single-stage repair of IAA with VSD [28]; if we take into consideration the cut-off value of 6.5 mm for the aortic root size our patient falls into the high-risk group and will require a close follow-up in order to promptly identify progressive obstruction.

Furthermore, a case of middle cerebral artery aneurysm rupture occurring at 2 weeks after undergoing congenital heart anomaly repair in a neonate with IAA has been published [29]. Also, another unusual complication following cardiac surgery for IAA was reported in an 8-year-old patient: giant aortic arch aneurysm [30], pointing to the fact that an extended period of follow-up is essential.

As expected, our patient had an association between 22q11.2DS and IAA type B, whereas IAA type A is found rarely in combination with this genetic deletion [31,32,33]; Paolo Volpe’s study confirms that IAA type B is usually syndromic (66.7% of the cases analyzed were associated with 22q11.2DS), this suggesting that IAA type A and type B could be two separate pathogenetic entities [1]. One possible explanation might lie in the disparate embryological origin of the different segments composing the aortic arch. On the other hand, in our patient, IAA was not the only cardiac malformation and what we considered relevant was to raise awareness on its association with other anomalies which makes the case even more difficult to approach.

Aditionally, recent studies have found that 22q11.2 deletion confers elevated risk for neuropsychiatric (including schizophrenia) and developmental disorders, including attention-deficit/hyperactivity disorder (ADHD), autism spectrum disorder (ASD) and intellectual disability. Notably, compared to the 1–2% prevalence of ASD in the general population, ASD has been reported to occur in approximately 23–45% of 22q11.2DS cases [34,35,36,37]. Regarding our case, these findings were all discussed with the parents at the time of genetic counselling. Consequently, we reinforced the need for close monitoring after birth of neurodevelopment with regard to both developmental delays and autism risk.

## 4. Conclusions

Our case brings into attention the association between a complex of severe cardiac anomalies and 22q11.2 microdeletion offering a clear and complete image starting from the prenatal diagnosis, continuing throughout the pregnancy and finally assessing in detail the modalities of post-natal medico-surgical treatment over a course of 7 weeks. 

We emphasize the importance of early correct diagnosis and increased awareness of extended panels of prenatal screening with NIPT that can be recommended to patients, the need for appropiate counselling of the parents and further managing the case in a multidisciplinary team in order to optimize the outcome.

## Figures and Tables

**Table 1:** Perinatal characteristics of the patient.

Case Highlights	Details
Maternal age	34 years
Gestational age at cardiologic diagnosis	22 weeks
Cardiologic diagnosis	IAA type B, malalignment-type VSD, pulmonary valve dyplasia, ARSA
Genetic diagnosis (amniocentesis with microarray)	22q11.2 deletion
Other ultrasonographic findings	Polyhydramnios Enlarged CSP Thymus hypoplasia Bulbous nose
Delivery route	Vaginal
Birthweight	3420 g
Postnatal echocardiography	Prenatal diagnosis confirmed
Heart surgery	Performed at 7 days of life Median sternotomy Extended latero-terminal anastomosis of the aortic arch and closure of VSD with bovine pericardial patch, closure of the patent ductus arteriosus Prophylactic antibiotics+ anticoagulants for 10 days
Total lentgh of stay in the cardiology unit	3 weeks
Recommendations at discharge	Sternal precautions for 10 weeks One year prophylactic therapy with ASA + endocarditis prophylaxis

IAA, interrupted aortic arch; VSD, ventricular septal defect; ARSA, aberrant right subclavian artery; CSP, cavum septum pellucidum; ASA, acetylsalicylic acid.

## Data Availability

Data sharing not applicable.

## Supplementary Materials

The following supporting information can be downloaded at: https://www.mdpi.com/article/10.3390/medicina59101838/s1, Video S1: Characteristic appearance of IAA, VSD, dilated pulmonary artery, ARSA and severe thymus hypoplasia.
